# Association between diabetes at different diagnostic ages and risk of cancer incidence and mortality: a cohort study

**DOI:** 10.3389/fendo.2023.1277935

**Published:** 2023-10-11

**Authors:** Yu Peng, Fubin Liu, Peng Wang, Yating Qiao, Changyu Si, Xixuan Wang, Jianxiao Gong, Huijun Zhou, Fengju Song, Fangfang Song

**Affiliations:** Department of Epidemiology and Biostatistics, Key Laboratory of Molecular Cancer Epidemiology, Key Laboratory of Prevention and Control of Major Diseases in the Population, Ministry of Education, National Clinical Research Center for Cancer, Tianjin Medical University Cancer Institute and Hospital, Tianjin Medical University, Tianjin, China

**Keywords:** diabetes, age at diagnosis, cancer incidence, cancer mortality, UK Biobank

## Abstract

**Background:**

Different ages for diagnosis of diabetes have diverse effects on risks of cardiovascular disease, dementia, and mortality, but there is little evidence of cancer. This study investigated the relationship between diabetes at different diagnostic ages and risks of cancer incidence and mortality in people aged 37–73 years.

**Methods:**

Participants with diabetes in the UK Biobank prospective cohort were divided into four groups: ≤40, 41–50, 51–60, and >60 years according to age at diagnosis. A total of 26,318 diabetics and 105,272 controls (1:4 randomly selected for each diabetic matched by the same baseline age) were included. We calculated the incidence density, standardized incidence, and mortality rates of cancer. Cox proportional hazard model was used to examine the associations of diabetes at different diagnostic ages with cancer incidence and mortality, followed by subgroup analyses.

**Results:**

Compared to corresponding controls, standardized incidence and mortality rates of overall and digestive system cancers were higher in diabetes diagnosed at age 41–50, 51–60, and >60 years, especially at 51–60 years. Individuals diagnosed with diabetes at different ages were at higher risk to develop site-specific cancers, with a prominently increased risk of liver cancer since the diagnosis age of >40 years. Significantly, participants with diabetes diagnosed at 51–60 years were correlated with various site-specific cancer risks [hazard ratio (HR) for incidence: 1.088–2.416, HR for mortality: 1.276–3.269]. Moreover, for mortality of digestive system cancers, we observed an interaction effect between smoking and diabetes diagnosed at 51–60 years.

**Conclusion:**

Our findings highlighted that the age at diagnosis of diabetes, especially 51–60 years, was critical risks of cancer incidence and mortality and may represent a potential preventative window for cancer.

## 1 Introduction

Due to the rapid development of the socio-economy and dramatic change of lifestyle, cancer incidence and mortality are increasing greatly, causing a huge disease burden worldwide (1, 2). In the world, it has been estimated that the new cancer cases and deaths were 19.3 million and 10 million in 2020, respectively (1). A number of well-known risk factors have been identified for cancer, some of which are also common for diabetes, such as age, obesity, and unhealthy lifestyles (3, 4). In 2021, 537 million adults (20–79 years) worldwide suffered from diabetes, causing over 6.7 million deaths (5). A growing body of convincing evidence indicates that diabetes has been associated with an increased risk of cancer incidence and mortality including digestive system cancers, bladder cancer, endometrial cancer, and so forth (6–9).

Numerous studies have examined the relationship of diabetes at different diagnostic ages with cardiovascular disease (CVD), dementia, and all-cause or CVD mortality (10–16) and elucidated that the risk of disease and mortality varies by age at diabetes diagnosis, revealing the importance of diabetes at different diagnostic ages on these associations. Although studies have investigated the relationship between diabetes and the risk of cancer incidence and mortality (6, 7), they have not yet explored whether diabetes at different diagnostic ages exert effect on cancer risk. At present, only a small number of researches illustrate that the risk of pancreatic cancer ranks highest when diabetes was diagnosed at 20–54 years (17), but there is no clear consensus. Additionally, people aged 45–64 years have a high incidence rate of diabetes, and this period is a critical period in the life to prevent cancer (18). Exploring the association between diabetes at different diagnostic ages and multiple cancer risks may be beneficial in providing ideas for effective measures to prevent specific cancers in specific diagnostic ages, especially considering that there are common and modifiable risk factors between diabetes and cancer.

Therefore, we aimed to explore the relationship between diabetes at different diagnostic ages and risks of cancer incidence and mortality in a large prospective cohort study by dividing the age of diagnosis into four groups: ≤40, 41–50, 51–60, and >60 years. This better understanding of the importance of diabetes at different diagnostic ages for cancer outcomes is necessary to improve disease management, determine high-quality care for individuals with diabetes, and identify the population at high risk of cancer.

## 2 Materials and methods

### 2.1 Study design and participants

The UK Biobank is a large prospective cohort study that recruited over 500,000 participants aged 37–73 years between 2006 and 2010 from 22 assessment centers in the United Kingdom, covering a variety of different settings to provide socioeconomic, ethnic heterogeneity and urban–rural mix (detailed information on study design, implementation, and data acquisition can be found at https://www.ukbiobank.ac.uk) (19). The baseline information of participants, such as sociodemographic characteristics, lifestyle, disease, and medical history, was obtained by a self-administered touchscreen questionnaire, brief verbal interview, and physical measurements filled in during the 2006–2010 recruitment. All participants provided written consent, and the UK Biobank study protocol was approved by the North West Multicenter Research Ethics Committee.

**Figure 1:**
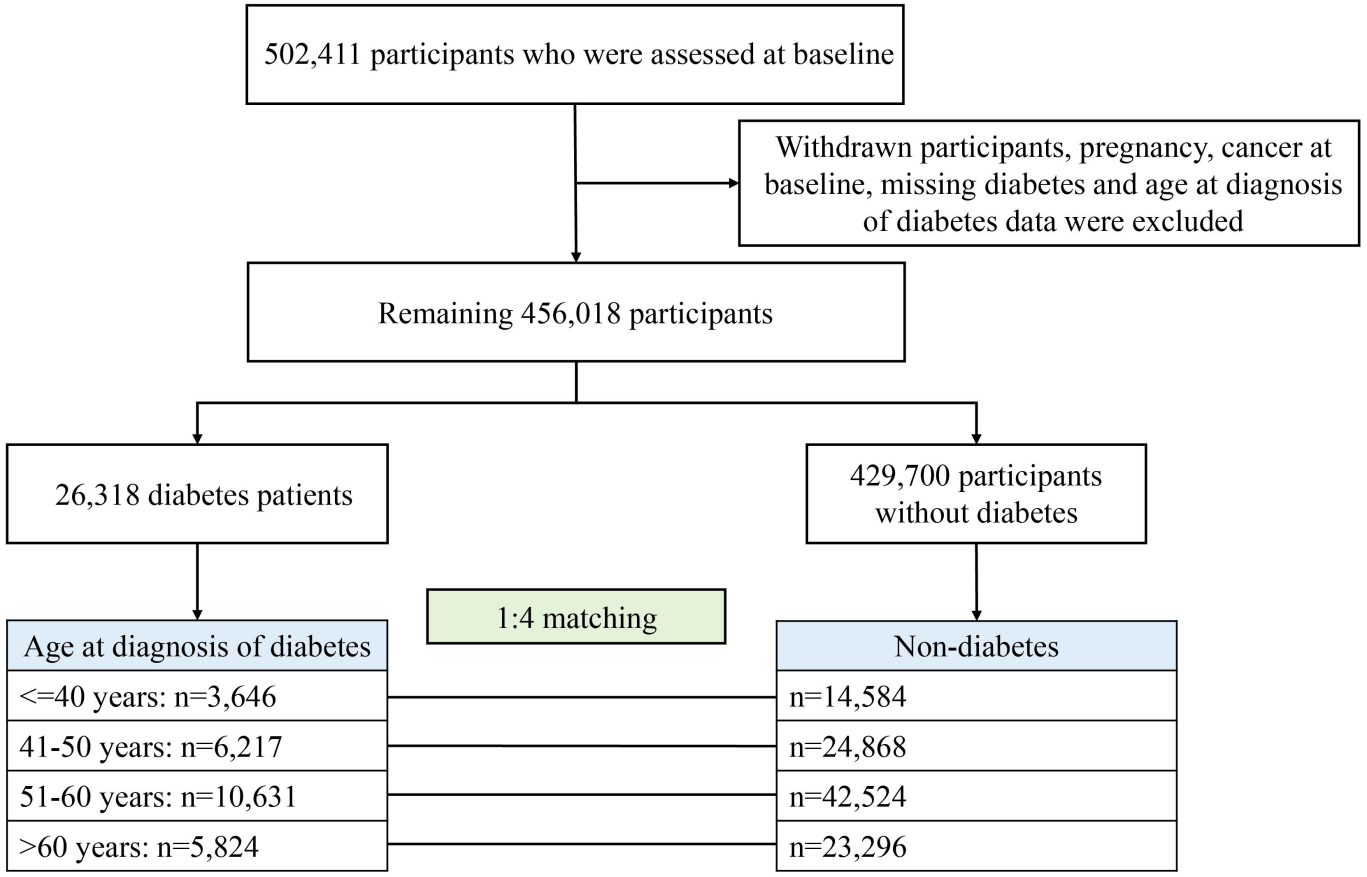
Study flow diagram.

For the present study, after excluding withdrawal, pregnancy, cancer diagnosed at baseline, and missing age at diagnosis of diabetes, 456,018 participants remained, of whom 26,318 had diabetes divided into four groups according to the age of diagnosis: ≤40, 41–50, 51–60, and >60 years (**Figure 1**). Each participant with diabetes was randomly matched with four controls of the same age at baseline from the remaining 429,700 participants without diabetes (i.e., for each patient with diabetes to be diagnosed at a certain age, his/her matched control was non-diabetes at that age, ensuring comparability between comparison groups). Finally, for four age groups for diabetes diagnosis, 14,584, 24,868, 42,524, and 23,296 participants were included as controls, respectively.

### 2.2 Ascertainment of diabetes and age at diagnosis

The baseline diabetes status was defined by at least one of the following criteria: (1) self-reported history of diabetes or reported a diagnosis of diabetes by a doctor; (2) hospital diagnosis of diabetes by the International Classification of Diseases, Ninth Revision (ICD-9: 250) and Tenth Revision (ICD-10: E10-E14); (3) use of diabetes medications or insulin; (4) glycosylated hemoglobin (HbA1c) levels ≥48 mmol/mol (20). The diagnosis age of diabetes can be directly obtained through self-reported age of diagnosis, inpatient records. For diabetes diagnosed only by HbA1c, the age of diabetes diagnosis was the baseline age. Those without the age of diabetes diagnosis were considered missing (1,644 individuals).

### 2.3 Outcome ascertainment

The incidence and mortality of overall (all cancers except non-melanoma skin cancer) and 24 site-specific cancers (first malignant cancer) were outcomes of interest. The cancer registration and death data were provided by National Health Service (NHS) England for participants in England and Wales, and NHS Central Register for participants in Scotland (https://biobank.ctsu.ox.ac.uk/crystal/refer.cgi?id=115559), and cancer registration data were obtained through linkage to the national cancer registries (https://biobank.ndph.ox.ac.uk/~bbdatan/CancerSummaryReport.html). Hospital admission data were obtained from the Health Episode Statistics and Scottish Morbidity Records. The detailed definition was captured using ICD-10, ICD-9, or self-reported data (**Supplementary Table S1**). The follow-up time of cancer incidence or cancer mortality outcome was expressed in person years (PYs) and calculated from baseline to the first occurrence of cancer cases or cancer deaths, or the end of the study period (the hospital admission and mortality data were available until 30 September 2021 and 31 October 2021, respectively). The median follow-up was 12.4 years.

### 2.4 Covariates

Variables included sex, race (White, Asian, Black, Mixed, and Other), body mass index (BMI; <25.0, 25.0–29.9, and ≥30 kg/m^2^), income (<18,000, 18,000–30,999, 31,000–51,999, and ≥52,000 pound), education (college or university, vocational, upper secondary, lower secondary, and others), socioeconomic status (defined by the Townsend deprivation index at recruitment), alcohol frequency (daily or almost daily, 3 or 4 times a week, 1 or 2 times a week, 1 to 3 times a month, special occasions only, never), smoking status (never, previous, and current), activity (low, moderate, and high), diet score (0, 1, 2, 3, and 4), family history of cancer, and the number of long-term conditions (none, one, two, three, four, five, and more) (21). The definition of a healthy diet score was shown in **Supplementary Table S2**. Missing values of covariates (29.32%–31.52%) were treated as dummy variables to preserve a large sample size and control the impact of missing data.

### 2.5 Statistical analysis

Continuous variables were expressed as mean ± standard deviation (SD), and were examined using Student’s t-tests. Categorical variables were presented as the quantity and percentage and analyzed using χ^2^ test.

The multivariable Cox proportional hazard model was used to examine hazard ratios (HRs) and 95% confidence intervals (CIs) for the associations of diabetes at different diagnostic ages with incidence and mortality of overall and individual cancers. The proportional hazards assumption was tested by Schoenfeld residuals, and no statistically significant deviations were observed. We calculated the incidence rates (per 10,000 person years) and mortality of overall and digestive system cancers among participants with diabetes versus control across different age groups. Moreover, standardized incidence rates (SIRs) of overall and digestive system cancers were calculated by dividing the observed number of cancer cases in diabetes at different diagnostic ages by the expected number of cancer cases in the corresponding control groups. The algorithm for standardized mortality rates (SMRs) was similar. In addition, we conducted a subgroup analysis to assess potential effect modification by sex, race, BMI, activity, smoke, alcohol, and diet score on the association between diabetes onset age at 51–60 years and the primary outcome of digestive system cancers. Then, the independent subgroup variable and its product term with age at diagnosis of diabetes were included in the multivariable Cox proportional hazard model to examine the multiplicative interaction.

To test the robustness of our results, we conducted a sensitivity analysis to exclude the outcome events that occurred during the first year of follow-up to avoid inverse causal relationships. In addition, we considered competing risk by using the Fine-Gray model to examine the association of diabetes at different diagnostic ages with incidence and mortality risks of cancer. All analyses were performed using SAS, version 9.4 (SAS Institute Inc, Cary, NC). A level of < 0.05 for two-sided *P* values was considered statistically significant.

## 3 Results

### 3.1 Characteristics of the study participants

A total of 131,590 participants were investigated in this study (**Figure 1**), and participants’ baseline characteristics were shown in **Table 1**. For most of the diagnostic age groups, in comparison to participants without diabetes at baseline, those diagnosed with diabetes were more likely to be male, smokers and less active, have higher BMI but lower education, income, socioeconomic status and diet score, and be with higher proportion of other diseases but slightly lower proportion of family history of cancer.

**Table 1:** Baseline characteristics of participants by age at diagnosis of diabetes.

Baseline characteristics		Age groups of diabetes diagnosis and its corresponding controls							
		≤ 40 years		41–50 years		51–60 years		> 60 years	
	Total	Non- diabetes	Diabetes	Non- diabetes	Diabetes	Non- diabetes	Diabetes	Non- diabetes	Diabetes
**N**	131588	14582	3646	24868	6217	42524	10631	23296	5824
**Age (y)**	131588	53.30 ± 8.55	53.31 ± 8.55	54.40 ± 6.84	54.40 ± 6.84	60.91 ± 4.42	60.91 ± 4.42	65.81 ± 2.42	65.81 ± 2.42
**Sex**	131588		*		*		*		*
Women	131588	7987 (54.77)	1378 (37.79)	13748 (55.28)	2113 (33.99)	23009 (54.11)	3796 (35.71)	12185 (52.31)	2261 (38.82)
Men		6595 (45.23)	2268 (62.21)	11120 (44.72)	4104 (66.01)	19515 (45.89)	6835 (64.29)	11111 (47.69)	3563 (61.18)
**Race**	130855		*		*		*		*
White		13680 (93.81)	2967 (81.38)	23505 (94.52)	4957 (79.73)	41091(96.63)	9342 (87.88)	22672 (97.32)	5347(91.81)
Other		820 (5.63)	654 (17.94)	1238 (4.98)	1208 (19.43)	1247 (2.93)	1185 (11.15)	503 (2.16)	439 (7.54)
Missing		82 (0.56)	25 (0.69)	125 (0.50)	52 (0.84)	186 (0.44)	104 (0.98)	121 (0.52)	38 (0.65)
**Body mass index (kg/m^2^)**	130742		*		*		*		*
< 25		5243 (35.96)	745 (20.43)	8814 (35.44)	573 (9.22)	13967 (32.84)	956 (8.99)	7332 (31.47)	615 (10.56)
25–29		6118 (41.96)	1234 (33.85)	10489 (42.18)	1889 (30.38)	19020(44.73)	3635 (34.19)	10839 (46.53)	2174 (37.33)
>= 30		3145 (21.57)	1593 (43.69)	5427 (21.82)	3673 (59.08)	9332 (21.95)	5929 (55.77)	5008 (21.50)	2992 (51.37)
Missing		76 (0.52)	74 (2.03)	138 (0.55)	82 (1.32)	205 (0.48)	111 (1.04)	117 (0.50)	43 (0.74)
**Education**	128701		*		*		*		*
College or university		5136 (35.22)	1043 (28.61)	8687 (34.93)	1530 (24.61)	12949 (30.45)	2459(23.13)	5818 (24.97)	1081 (18.56)
Vocational		1580 (10.84)	400 (10.97)	2730 (10.98)	844 (13.58)	5596 (13.6)	1592 (14.98)	3232 (13.87)	894 (15.35)
Upper secondary		1717 (11.77)	359 (9.85)	2991 (12.03)	655 (10.54)	4301 (10.11)	932 (8.77)	1906 (8.18)	431 (7.40)
Lower secondary		4021 (27.58)	987 (27.07)	6765 (27.20)	1749 (28.13)	10025 (23.57)	2416(22.73)	5158 (22.14)	1167 (20.04)
Others		1843 (12.64)	747 (20.49)	3218 (12.94)	1264 (20.33)	8815 (20.73)	2916 (27.43)	6654 (28.56)	2093 (35.94)
Unknown		285 (1.95)	110 (3.02)	477 (1.92)	175 (2.81)	838 (1.97)	316 (2.97)	528 (2.27)	158 (2.71)
**Income (pound)**	109297		*		*		*		*
< 18,000		2266 (15.54)	1018 (27.92)	3727 (14.99)	1668 (26.83)	9034 (21.24)	3096 (29.12)	6804 (29.21)	2126 (36.50)
18,000–30,999		2933 (20.11)	751 (20.60)	4886 (19.65)	1352 (21.75)	10088 (23.72)	2490 (23.42)	6170 (26.49)	1425 (24.47)
31,000–51,999		3604 (24.72)	683 (18.75)	6130 (24.65)	1171 (18.84)	8754 (20.59)	1813 (17.05)	3585 (15.39)	681 (11.69)
> 52,000		3792 (26.10)	544 (14.92)	6708 (26.97)	979 (15.75)	7464 (17.55)	1283(12.07)	1918 (8.23)	354 (6.08)
Missing		1987 (13.63)	650 (17.83)	3417 (13.74)	1047 (16.84)	7184 (16.89)	1949 (18.33)	4819 (20.69)	1238 (21.26)
**Socioeconomic status**	131440	−1.31 ± 3.07	0.12 ± 3.59 *	−1.38 ± 3.04	0.09 ± 3.50 *	−1.57 ± 2.93	−0.55 ± 3.38 *	−1.65 ± 2.91	−0.82 ± 3.27 *
**Alcohol frequency**	131220		*		*		*		*
Daily or almost daily		2883 (19.77)	519 (14.23)	5054 (20.32)	714 (11.48)	9842 (23.14)	1612 (15.16)	5679 (24.38)	996 (17.10)
3 or 4 times a week		3463 (23.75)	539 (14.78)	6012 (24.18)	803 (12.92)	10148 (23.86)	1718 (16.16)	5090 (21.85)	996 (17.10)
1 or 2 times a week		3870 (26.54)	837 (22.96)	6553 (26.35)	1431 (23.02)	10468 (24.62)	2466 (23.20)	5529 (23.73)	1370 (23.52)
1 to 3 times a month		1706 (11.70)	414 (11.35)	2789 (11.22)	873 (14.04)	4256 (10.01)	1287 (12.11)	2211 (9.49)	628 (10.78)
Special occasions only		1562 (10.71)	636 (17.44)	2649 (10.65)	1212 (19.49)	4612 (10.85)	1905 (17.92)	2808 (12.05)	982 (16.86)
Never		1057 (7.25)	689(18.09)	1743 (7.01)	1152 (18.54)	3119 (7.33)	1583 (14.89)	1929 (8.28)	826 (14.18)
Missing		41 (0.28)	12 (0.33)	68 (0.27)	32 (0.51)	79 (0.19)	60 (0.56)	50 (0.21)	26 (0.45)
**Smoking status**	130721		*		*		*		*
Never		8305 (56.95)	2004 (54.96)	14260 (57.34)	3096 (49.80)	22524 (52.97)	4550 (42.80)	11744 (50.41)	2320 (39.84)
Previous		4579 (31.40)	1135 (31.13)	7822 (31.45)	2193 (35.27)	16042 (37.72)	4823 (45.37)	9684 (41.57)	2887 (49.57)
Current		1625 (11.14)	480 (13.17)	2660 (10.70)	866 (13.93)	3723(8.76)	1144 (10.76)	1691 (7.26)	564 (9.68)
Missing		73 (0.50)	27 (0.74)	126 (0.51)	62 (1.00)	235 (0.55)	114 (1.07)	177 (0.76)	53 (0.91)
**Activity**	104488		*		*		*		*
Low		2175 (14.92)	805 (22.08)	3817 (15.35)	1447 (23.27)	5926 (13.94)	2301 (21.64)	2739 (11.76)	1064 (18.27)
Moderate		4827 (33.10)	1092 (29.95)	8233 (33.11)	1882 (30.27)	14146 (33.27)	3298 (31.02)	7685 (32.99)	1852 (31.80)
High		4904 (33.63)	945 (25.92)	8174 (32.87)	1473 (23.69)	13777 (32.40)	2638 (24.81)	7750 (33.27)	1538 (26.41)
Missing		2676 (18.35)	804 (22.05)	4644 (18.67)	1415 (22.76)	8675 (20.40)	2394 (22.52)	5122 (21.99)	1370 (23.52)
**Diet score**	124474		*		*		*		*
0		2046(14.03)	531 (14.56)	3416(13.74)	995 (16.00)	5118 (12.04)	1581 (14.87)	2813 (12.08)	801 (13.75)
1		4208 (28.86)	1140 (31.27)	7201 (28.96)	1889 (30.38)	12012 (28.25)	3275 (30.81)	6620 (28.42)	1810 (31.08)
2		4097 (28.10)	963 (26.41)	7056 (28.37)	1656 (26.64)	12413 (29.19)	2898 (27.26)	6738 (28.92)	1658 (28.47)
3		2650 (18.17)	512 (14.04)	4545 (18.28)	881 (14.17)	8126 (19.11)	1642 (15.45)	4402 (18.90)	890 (15.28)
4		891 (6.11)	181 (4.96)	1497 (6.02)	252 (4.05)	2822 (6.64)	493 (4.64)	1475 (6.33)	280 (4.81)
**Family history of cancer**	103367	4794 (32.88)	1085 (29.76) *	8479 (34.10)	1861 (29.93) *	15863 (37.30)	3649 (34.32) *	8809 (37.81)	2083 (35.77) *
**No. of long-term conditions**	131588		*		*		*		*
None		6289 (43.13)	774 (21.23)	10556(42.45)	1191 (19.16)	14574 (34.27)	1604 (15.09)	6373 (27.36)	746(12.81)
One		4837 (33.17)	1259 (34.53)	8282 (33.30)	2205 (35.47)	14811 (34.83)	3672 (34.54)	8205 (35.22)	1997 (34.29)
Two		2258 (15.48)	863 (23.67)	3925 (15.78)	1492 (24.00)	8241 (19.38)	2846 (26.77)	5310 (22.79)	1632 (28.02)
Three		784 (5.38)	421 (11.55)	1409 (5.67)	776 (12.48)	3257 (7.66)	1492 (14.03)	2263 (9.71)	854 (14.66)
Four		279 (1.91)	178 (4.88)	466 (1.87)	329 (5.29)	1119 (2.63)	637 (5.99)	787 (3.38)	393 (6.75)
Five and more		135 (0.93)	151 (4.14)	230 (0.92)	224 (3.60)	522 (1.23)	380 (3.57)	358 (1.54)	202 (3.47)

Data are mean ± SD, or N (%).

*Refers to significant difference between diabetes and non-diabetes at different ages of diagnosis. T-test was used to test the difference of continuous variables between hypertensive participants and controls and χ^2^ for categorical variables.

### 3.2 Absolute and relative risk of cancer onset and death across different ages at diagnosis

Except for the diagnosis age ≤40 years, the participants with diabetes had a higher proportion of overall and multiple cancers, mainly digestive system cancers (esophageal, gastric, colorectal, and liver cancers), bladder and endometrial cancers, during follow-up period compared with the participants without diabetes (**Supplementary Table S3**). Furthermore, compared with those without diabetes, patients with diabetes diagnosed at 51–60 years had higher proportion of deaths from 10 specific cancers, particularly digestive system cancers. In other diagnosis age groups, there were fewer types of cancers with higher mortality in diabetes (**Supplementary Table S4**).

**Figure 2:**
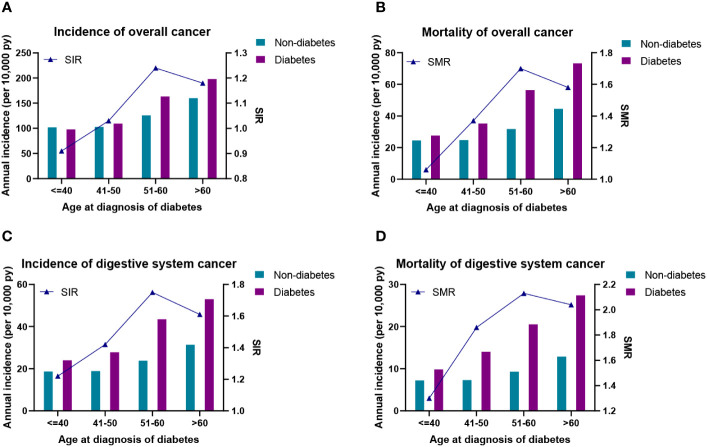
**(A)** Incidence density and standardized incidence of overall cancer. **(B)** Incidence density and standardized mortality of overall cancer. **(C)** Incidence density and standardized incidence of digestive system cancer. **(D)** Incidence density and standardized mortality of digestive system cancer. SIR, standardized incidence rate; SMR, standardized mortality rate.

The higher annual incidence (109.22–198.09 per 10,000 PYs) and mortality (35.26–73.22 per 10,000 PYs) of overall cancer were observed in those with age of diabetes diagnosed at 41–50, 51–60, and >60 years than their counterparts (**Figures 2A, B**). Specially, compared with their controls, the annual incidence (23.99–53.00 per 10,000 PYs) and mortality (9.82–27.40 per 10,000 PYs) of digestive system cancers were significantly increased in all diagnostic age groups (**Figures 2C, D**). Furthermore, the relative risks of overall cancer, digestive system cancers, and their mortality were also presented as SIRs and SMRs in **Figure 2**. The SIR of overall cancer was higher than that of the corresponding control groups except for the diagnosis age ≤40 years (**Figure 2A**). For different ages at diabetes diagnosis, the SMR of overall cancer and SIR/SMR of digestive system cancers were higher than those in the corresponding control groups (**Figures 2B–D**), remarkably patients with diabetes at diagnosis age 51–60 years (SIR: overall cancer, 1.24; SMRs: overall cancer, 1.70; digestive system cancers, 2.13).

### 3.3 Diabetes at different diagnostic ages and risk of cancer

**Figure 3:**
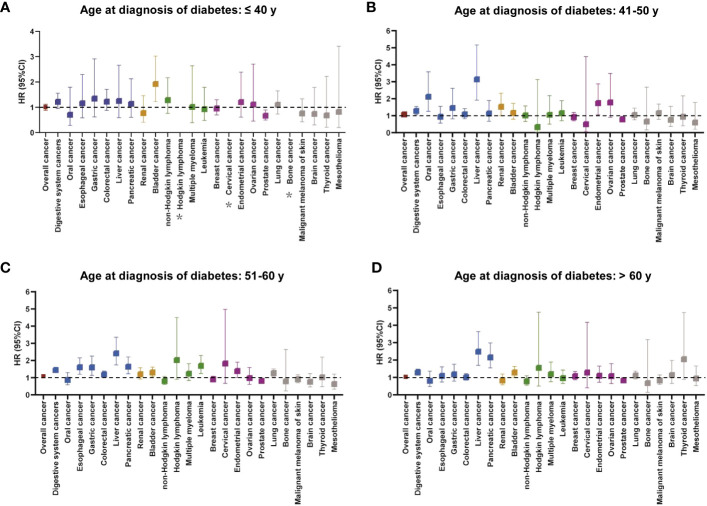
The relationship between cancer incidence risk and diabetes at different diagnostic ages (**A**: ≤ 40 years, **B**: 41–50 years, **C**: 51–60 years, and **D**: > 60 years). The colors represented different body systems. Multivariable analysis was adjusted for sex, race, income, education, alcohol consumption, physical activity, smoking, body mass index, diet score, family history of cancer, and long-term conditions. *HR was not calculated due to small sample size. CI, confidence interval; HR, hazard ratio.

The relationship between diabetes at different diagnostic ages and cancer risk was presented in **Figure 3** and **Supplementary Table S5**. Overall, in comparison to the corresponding controls, only patients with diabetes with diagnosis age 51–60 years had an increased risk of overall cancer after adjusting for relevant factors [HR (95% CI): 1.088 (1.029–1.152)]. For risk of site-specific cancers, diabetes with diagnosis age ≤40 years was only associated with an increased risk of bladder cancer (**Figure 3A**). At diagnosis age of 41–50 years, risks of four cancers (oral, liver, kidney, and endometrial cancers) were elevated in patients with diabetes (**Figure 3B**). Surprisingly, those with diagnosis age at 51–60 years significantly increased risks of multiple cancers, mainly digestive system cancers, including esophageal cancer [HR (95% CI): 1.605 (1.197–2.152)], gastric cancer [HR (95% CI): 1.600 (1.133–2.258)], liver cancer [HR (95% CI): 2.416 (1.743–3.349)] and pancreatic cancer [HR (95% CI): 1.642 (1.222–2.205)] (**Figure 3C**). With respect to diagnosis age of >60 years, we observed patients with diabetes were susceptible to developing into three types of cancers, namely, liver, pancreatic, and bladder cancers (**Figure 3D**). In contrast, there was a significant decrease risk of prostate cancer across all age groups, prominently lowest in the youngest age group [HR (95% CI): 0.668 (0.499–0.894)].

### 3.4 Diabetes at different diagnostic ages and risk of cancer mortality

**Figure 4:**
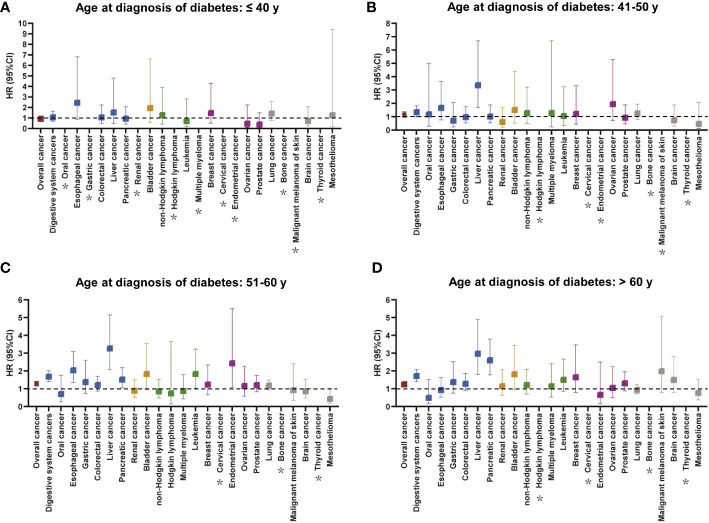
The relationship between cancer mortality risk and diabetes at different diagnostic ages (**A**: ≤ 40 years, **B**: 41–50 years, **C**: 51–60 years, **D**: > 60 years). The colors represented different body systems. Multivariable analysis was adjusted for sex, race, income, education, alcohol consumption, physical activity, smoking, body mass index, diet score, family history of cancer, no. of long-term conditions, antihypertensive, and lipid-lowering drugs. *HR was not calculated due to small sample size. CI, confidence interval; HR, hazard ratio.

In different diagnosis age groups, individuals with diabetes had a lower survival probability for overall cancer or digestive system cancers than those without diabetes, especially among participants diagnosed at 51**–**60 years and >60 years (**Supplementary Figure S1**). The findings for the associations between diabetes at different diagnostic ages and cancer mortality were shown in **Figure 4** and **Supplementary Table S6**. After adjusting for relevant factors, the increased risk of overall cancer mortality in patients with diabetes compared with matched controls was observed at diagnosis age of 51–60 and >60 years, but not ≤40 and 41–50 years. For site-specific cancers, there were no statistically significant relationships between diabetes diagnosed at ≤40 years and individual cancers (**Figure 4A**). Patients with diabetes diagnosed at 41–50 years only had a higher risk of liver cancer mortality compared with the corresponding controls (**Figure 4B**). Five cancers had increased risk of mortality in diabetes compared with non-diabetes at diagnosis age of 51–60 years, that is, esophageal cancer [HR (95% CI): 2.037 (1.343–3.089)], liver cancer [HR (95% CI): 3.269 (2.073–5.154)], pancreatic cancer [HR (95% CI): 1.519 (1.057-2.183)], leukemia [HR (95% CI): 1.834 (1.041–3.229)], and endometrial cancer [HR (95% CI): 2.436 (1.079–5.501)] (**Figure 4C**). Meanwhile, diabetes diagnosed at >60 years demonstrated a positive association with mortality risk of liver cancer and pancreatic cancer (**Figure 4D**).

### 3.5 Subgroup analysis

Since we mainly found that the incidence and mortality risks of digestive system cancers were elevated in patients with diabetes at diagnosis age of 51–60 years, we conducted a subgroup analysis based on baseline characteristics and lifestyles (**Table 2**). Diabetes was positively related to the risks of digestive system cancers incidence and mortality in both genders and White persons. Moreover, both the risks of digestive system cancer incidence and mortality were increased in diabetic patients with BMI ≥25 kg/m^2^ and were even higher in those with unhealthy lifestyles such as smoking, alcohol consumption, lower physical activity, and diet score. There was an interaction effect between smoke and diabetes at diagnosis age of 51–60 years on mortality of digestive system cancers (*P* for interaction: 0.027).

**Table 2:** Subgroup analysis for incidence and mortality risks of digestive system cancers in patients with diabetes at diagnosis age of 51–60 years by baseline characteristics and lifestyle.

	Incidence of digestive system cancer		Mortality of digestive system cancer	
	HR (95% CI)	*P* for interaction	HR (95% CI)	*P* for interaction
**Sex**		0.822		0.745
Female	1.432 (1.154–1.776)		1.718 (1.235–2.390)	
Male	1.441 (1.262–1.646)		1.762 (1.439–2.158)	
**Race**		0.270		0.248
White	1.457 (1.298–1.635)		1.783 (1.497–2.125)	
Others	1.214 (0.681–2.164)		1.120 (0.427–2.935)	
**Body mass index**, kg/m^2^		0.984		0.351
< 25	1.507 (1.050–2.162)		1.367 (0.774–2.413)	
≥ 25	1.445 (1.286–1.623)		1.832 (1.532–2.191)	
**Activity**		0.107		0.739
No	1.861 (1.464–2.365)		1.780 (1.249–2.538)	
Yes	1.392 (1.201–1.613)		1.623 (1.294–2.036)	
**Smoke**		0.143		0.027
No	1.320 (1.091–1.597)		1.338 (0.987–1.813)	
Yes	1.522 (1.320–1.754)		2.008 (1.622–2.486)	
**Alcohol**		0.311		0.107
No	1.227 (0.859–1.753)		1.146 (0.665–1.974)	
Yes	1.466 (1.302–1.650)		1.832 (1.530–2.195)	
**Diet score**		0.587		0.504
Low	1.480 (1.299–1.685)		1.781 (1.461–2.172)	
High	1.482 (1.141–1.926)		1.630 (1.079–2.464)	

CI, confidence interval; HR, hazard ratio.

The model of incidence was adjusted for sex, race, income, education, alcohol consumption, physical activity, smoking, body mass index, diet score, family history of cancer, and long-term conditions.

The model of mortality was adjusted for sex, race, income, education, alcohol consumption, physical activity, smoking, body mass index, diet score, family history of cancer, long-term conditions, antihypertensive, and lipid-lowering drugs.

### 3.6 Sensitivity analysis

In sensitivity analyses, after excluding participants with outcome event within the first year of follow-up (**Supplementary Tables S7 and S8**), the main results of the association between diabetes at different diagnostic ages and risks of cancer incidence and mortality were not affected, demonstrating the stability of our results. More importantly, it was found that the association between diabetes at different diagnostic ages and risks of cancer incidence and cancer mortality was also stable through competitive risk (**Supplementary Tables S9, S10**).

## 4 Discussion

In this large and prospective cohort study, we observed higher absolute and relative risks of overall cancer and digestive system cancers in individuals with diabetes diagnosed at different ages. Diabetes diagnosed at different ages suffered from high risk to develop specific cancers, with a dominantly increased risk of liver cancer throughout all the diagnosis age of diabetes at ≥40 years. Notably, diabetes diagnosed at 51–60 years was associated with risks of multiple site-specific cancers, including digestive system cancers, lung cancer, and leukemia. Moreover, for the age of diagnosis 51–60 years, there was an interaction effect of smoking and diabetes on the mortality of digestive system cancers.

Emerging evidence have represented that, compared with those without diabetes, diabetes was associated with elevated risks of incidence and mortality of overall cancer and multiple cancers but a reduced incidence of prostate cancer (7, 22–26). A previous study using the UK Biobank database also illustrated that diabetes is associated with a higher risk of gastric, liver, lung, bladder, and endometrial cancers, but a decreased risk of prostate cancer (27), consistent with the findings in diabetes diagnosed at age 51–60 years in our study. However, we also observed increased risks of esophageal cancer, colorectal cancer, pancreatic cancer, and leukemia in patients with diabetes diagnosed at 51–60 years. Additionally, risks of oral cancer, liver cancer, and kidney cancer were significantly elevated for those at the diagnosis age of 41–50 years. These differences between the two studies may be due to the influence of age at diabetes diagnosis. A number of studies revealed that diabetes at different ages of diagnosis show various risks of CVD, dementia, mortality, and so forth (10–17). One of these studies depicted the risks of other adverse outcomes including CVD, disability, cognitive impairment, and all-cause mortality were related to diabetes at diagnosis of age 51–60 years (10), which was comparable to our study. To date, only one study from Chinese population reported a higher risk of pancreatic cancer in the younger age at diagnosis of diabetes (20–54 years) (17), but according to our findings, patients with diabetes diagnosed at 51–60 and >60 years had a higher risk of pancreatic cancer, compared with matched controls. This may be due to differences in race and classification of age groups for diabetes diagnosis.

At present, the underlying mechanisms for the association between the age at diagnosis of diabetes and risks of cancer incidence and mortality remains unclear. Accumulating evidences supported that insulin resistance, subsequent hyperinsulinemia, and hyperglycemia were hypothesized to be the biological mechanisms linking diabetes to cancer risk (28–33). Hyperinsulinemia can influence cancer development by indirectly affecting endogenous hormones, and hyperglycemia can induce oxidative stress and DNA damage, both of which regulate signaling pathways and stimulate cancer cell proliferation, thereby affecting cancer development and progression (28–33). The risk of cancer begins to increase among people aged 50–64 years (34, 35). Therefore, in the age of 51–60 years, compared with those without diabetes, the increased cancer risk of participants with diabetes may be ascribed to a synergistic effect between biological age and the influence of hyperglycemia, insulin resistance and hyperinsulinemia. Whereas, the observed inverse association between diabetes and prostate cancer can be explained by the lower levels of testosterone or leptin and insulin-like growth factor 1 in men with diabetes (36). For the diagnosis age ≤40 years and 41–50 years, there is no statistical significance between diabetes and multiple site-specific cancers, which may be caused by the lower statistical ability due to the fewer cases of diabetes at diagnosis age ≤50 years.

The highlights of this study indicated the importance of different ages at diagnosis for individuals with diabetes on risks of cancer incidence and mortality. In addition to the wide range of cancers that should be screened for individuals with diabetes diagnosed at 51–60 years, the remaining age groups should also be screened for their specific cancers. Moreover, individuals with diabetes, in each diagnosed age group, can reduce the risk of cancer by changing common and changeable risk factors between diabetes and cancer through healthy lifestyle. Researches demonstrated that adopting a healthy lifestyle cannot only reduce the risk of diabetes (37, 38) but also be associated with a significantly lower risk of cancer incidence and mortality (39). Five cohort studies and a meta-analysis indicated that adherence to a healthy lifestyle was associated with a lower risk of overall cancer and specific cancers (esophagus, lung, liver, colorectal, and renal) incidence or mortality in subjects with diabetes (40, 41). In particular, our research discovered that, when the age of diabetes diagnosis was 51–60 years old, smokers had a higher risk of digestive system cancers mortality. Therefore, in the population aged 51–60 years, both individuals with diabetes and non-diabetes should adhere to a healthy lifestyle of quit smoking to reduce the risk of cancer mortality.

Our study had several major strengths. As far as we know, this was the first study to comprehensively explore the association between diabetes at different diagnostic ages and risks of cancer incidence and mortality, with the advantages of large sample size and long follow-up time. In addition, by matching the baseline ages of the diabetes and non-diabetes groups, we ensured as much as possible that the controls did not have diabetes when individuals were diagnosed with diabetes and could ensure comparability between comparison groups. We also acknowledged some limitations of our study. First of all, we did not distinguish type 1 diabetes and type 2 diabetes in this study, because there was no detailed information at baseline for diabetes patients diagnosed by doctors or judged by HbA1C. Second, although the potential confounding factors have been meticulously adjusted, we could not exclude the residual confounding in our analysis. For example, it was difficult to assess the effect of cancer staging or diabetes drugs on the correlation between the age of diabetes diagnosis and cancer. Although setting the missing covariate as a dummy variable could preserve a larger sample size, it may overestimate the associations. Moreover, this study had some selection bias, such as immortal time bias, which may lead to fewer cancer outcomes in the exposed groups and the underestimation of the associations. We used strict inclusion and exclusion criteria and selected controls at the same matched age at baseline to minimize bias as much as possible. Finally, although we have excluded cancer patients who developed within 1 year after follow-up, we could not completely exclude the reverse causal relationship between the age at diabetes diagnosis and cancer.

### 4.1 Conclusions

In conclusion, these representative data indicated that individuals with diabetes diagnosed at different ages have high risks of specific cancers. Individuals with diabetes suffered a persistently increased risk of liver cancer since the diagnosis age of >40. It is worth noting that those diagnosed aged 51–60 years linked to risks of multiple site-specific cancers and smoking and diabetes have interaction on mortality of digestive system cancer. This emphasized that age at diagnosis and tobacco control is important windows for the management, screening, and preventative strategies of diabetes and cancer.

## Data availability statement

The original contributions presented in the study are included in the article/**Supplementary Material**, further inquiries can be directed to the corresponding authors.

## Ethics statement

The studies involving humans were approved by the North West Multicenter Research Ethics Committee. The studies were conducted in accordance with the local legislation and institutional requirements. The participants provided their written informed consent to participate in this study.

## Author contributions

YP: Writing – review & editing, Data curation, Formal Analysis, Investigation, Methodology, Visualization, Writing – original draft. FL: Data curation, Writing – review & editing, Validation. PW: Writing – review & editing, Formal Analysis. YQ: Formal Analysis, Writing – review & editing. CS: Formal Analysis, Writing – review & editing. XW: Formal Analysis, Writing – review & editing. JG: Writing – review & editing, Methodology. HZ: Methodology, Writing – review & editing. FJS: Writing – review & editing, Conceptualization, Funding acquisition, Project administration, Resources, Supervision. FFS: Conceptualization, Funding acquisition, Project administration, Resources, Supervision, Writing – review & editing.
