# Supplementary material for: Association between diabetes at different diagnostic ages and risk of cancer incidence and mortality: a cohort study
Source: Front Endocrinol (Lausanne). 2023 Oct 11;14:1277935. doi: 10.3389/fendo.2023.1277935 (PMC10600378; doi:10.3389/fendo.2023.1277935)
Supplement: Supplementary file 1 [file DataSheet_1.docx]

**Association between** **Diabetes at Different Diagnostic Ages and Risk of Cancer Incidence and Mortality: A Cohort Study in the UK Biobank**

**Supplementary Tables:**

Supplementary Table 1. Cancer definitions in UK Biobank study.

Supplementary Table 2. Definition of each component of a healthy diet score.

Supplementary Table 3. The distribution of cancer incidence in diabetes and non-diabetes at different ages of diagnosis.

Supplementary Table 4. The distribution of cancer mortality in diabetes and non-diabetes at different ages of diagnosis.

Supplementary Table 5. The relationship between diabetes at different diagnostic ages and cancer incidence risk.

Supplementary Table 6. The relationship between diabetes at different diagnostic ages and cancer mortality risk.

Supplementary Table 7. Association between diabetes at different diagnostic ages and cancer incidence risk after excluding participants with outcome events within the first year of follow-up.

Supplementary Table 8. Association between diabetes at different diagnostic ages and cancer mortality risk after excluding participants with outcome events within the first year of follow-up.

Supplementary Table 9. Association between diabetes at different diagnostic ages and cancer incidence risk: results from multivariate competing risk model.

Supplementary Table 10. Association between diabetes at different diagnostic ages and cancer mortality risk: results from multivariate competing risk model.

**Supplementary Figures:**

Supplementary Figure 1. Survival curves of overall cancer and digestive system cancer in different age groups diagnosed with diabetes.

**Supplementary Table 1. Cancer definitions in UK Biobank study.**

|  | **ICD-9** | **ICD-10** | **Self-reported** |
| --- | --- | --- | --- |
|  | **Field ID: 40013, 41271** | **Field ID: 41270, 40006, 40001, 40002,** | **Field ID: 20001** |
| **All cancer** | 140-208 (except 173) | C00-C97 (except C44) | 1001-1088 (except 1060-1062, 1073) |
| **Oral** | 140-149 | C00-C14 | 1004, 1005, 1010, 1011, 1012, 1015, 1016, 1077, 1078, 1079 |
| **Esophagus** | 150 | C15 | 1017 |
| **Stomach** | 151 | C16 | 1018 |
| **Colorectum** | 153-154 | C18-C20 | 1020, 1022, 1023, 1086 |
| **Liver** | 155 | C22 | 1024 |
| **Pancreas** | 157 | C25 | 1026, 1088 |
| **Lung** | 162 | C34 | 1001, 1027, 1028 |
| **Bone** | 170 | C40-C41 | 1063, 1085 |
| **Malignant melanoma** | 172 | C43 | 1059 |
| **Breast** | 174 | C50 | 1002 |
| **Cervix uteri** | 180 | C53 | 1041, 1072 |
| **Endometrium** | 182 | C54.1 | 1040 |
| **Ovary** | 1830 | C56 | 1039 |
| **Prostate** | 185 | C61 | 1044 |
| **Kidney** | 1890-1891 | C64-C65 | 1034 |
| **Bladder** | 188 | C67 | 1035 |
| **Brain** | 191 | C71 | 1032 |
| **Thyroid** | 193 | C73 | 1065 |
| **Non-Hodgkin lymphoma** | 202 | C82-C85 | 1053 |
| **Hodgkin lymphoma** | 201 | C81 | 1052 |
| **Multiple myeloma** | 203 | C90 | 1050 |
| **Leukemia** | 204-208 | C91-C95 | 1048 |
| **Mesothelioma** | / | C45 | 1064 |

Abbreviations: ICD, International Classification of Diseases

**Supplementary Table 2. Definition of each component of a healthy diet score.**

| **Diet score components** | **Goal (1 point)** | **Individual components included** | **One serving equals to** | **Field IDs** |
| --- | --- | --- | --- | --- |
| **Total vegetable and fruit intake** | ≥ 5 servings/day | Vegetable intake | 3 heaped tablespoons | 1289, 1299 |
|  |  | Fruit intake | 1 piece of fresh fruit  5 pieces of dried fruit | 1309, 1319 |
| **Whole grains intake** | ≥ 5.5 servings/day | Wholemeal or wholegrain bread intake | 1 slice | 1438, 1448 |
|  |  | Cereal (bran/oat/muesli) intake | 1 bowl | 1458, 1468 |
| **Red meat intake** | ≤ 1 serving/week | Beef intake | ≤ once/week | 1369 |
|  |  | Lamb intake | ≤ once/week | 1379 |
|  |  | Pork intake | ≤ once/week | 1389 |
| **Processed meat intake** | ≤ 1 serving/week | / | ≤ once/week | 1349 |

The Healthy eating score is the sum of the four food types and ranges from 0 to 4.

**Supplementary Table 3. The distribution of cancer incidence in diabetes and non-diabetes at different ages of diagnosis.**

|  | **≤ 40 years** | | **41-50 years** | | **51-60 years** | | **> 60 years** | |
| --- | --- | --- | --- | --- | --- | --- | --- | --- |
|  | **Non-diabetes, n (%)** | **Diabetes, n (%)** | **Non-diabetes, n (%)** | **Diabetes, n (%)** | **Non-diabetes, n (%)** | **Diabetes, n (%)** | **Non-diabetes, n (%)** | **Diabetes, n (%)** |
| **Overall cancer** | 1540 (10.56) | 398 (10.92) | 2664 (10.71) | 769 (12.37) * | 6417 (15.09) | 1906 (17.93) * | 4414 (18.95) | 1235 (21.21) * |
| **Oral cancer** | 36 (0.25) | 6 (0.16) | 65 (0.26) | 27 (0.43) * | 139 (0.33) | 37 (0.35) | 90 (0.39) | 20 (0.34) |
| **Esophageal cancer** | 35 (0.24) | 13 (0.36) | 64 (0.26) | 26 (0.42) * | 149 (0.35) | 88 (0.83) * | 108 (0.67) | 39 (0.67) * |
| **Gastric cancer** | 24 (0.16) | 11 (0.11) | 40 (0.16) | 23 (0.37) * | 117 (0.28) | 62 (0.58) * | 93 (0.40) | 39 (0.67) * |
| **Colorectal cancer** | 162 (1.11) | 54 (1.48) | 285 (1.15) | 92 (1.48) * | 800 (1.88) | 264 (2.48) * | 598 (2.57) | 171 (2.94) |
| **Liver cancer** | 23 (0.16) | 13 (0.36) | 38 (0.15) | 51 (0.82) * | 98 (0.23) | 85 (0.80) * | 71 (0.30) | 59 (1.01) * |
| **Pancreatic cancer** | 46 (0.32) | 16 (0.44) | 69 (0.28) | 26 (0.42) | 179 (0.42) | 80 (0.75) * | 121 (0.52) | 68 (1.17) * |
| **Lung cancer** | 119 (0.82) | 37 (1.01) | 209 (0.84) | 65 (1.05) | 587 (1.38) | 231 (2.17) * | 423 (1.82) | 146 (2.51) * |
| **Bone cancer** | 8 (0.05) | 4 (0.11) | 12 (0.05) | 3 (0.05) | 16 (0.04) | 4 (0.04) | 14 (0.06) | 2 (0.03) |
| **Malignant melanoma of skin** | 100 (0.69) | 15 (0.41) | 185 (0.74) | 39 (0.63) | 411 (0.97) | 85 (0.80) | 264 (1.13) | 49 (0.84) |
| **Renal cancer** | 53 (0.36) | 14 (0.38) | 83 (0.33) | 44 (0.71) * | 217 (0.51) | 91 (0.86) * | 150 (0.64) | 42 (0.72) |
| **Bladder cancer** | 57 (0.39) | 38 (1.04) * | 113 (0.45) | 48 (0.77) * | 347 (0.82) | 146 (1.37) * | 274 (1.18) | 107 (1.84) * |
| **Brain cancer** | 32 (0.22) | 7 (0.19) | 50 (0.20) | 11 (0.18) | 119 (0.28) | 23 (0.22) | 73 (0.31) | 18 (0.31) |
| **Thyroid cancer** | 20 (0.14) | 4 (0.11) | 27 (0.11) | 10 (0.16) | 40 (0.09) | 10 (0.09) | 19 (0.08) | 10 (0.17) |
| **Non-Hodgkin lymphoma** | 63 (0.43) | 23 (0.63) | 105 (0.42) | 36 (0.58) | 297 (0.70) | 66 (0.62) | 224 (0.96) | 45 (0.77) |
| **Hodgkin lymphoma** | 3 (0.02) | 3 (0.08) | 9 (0.04) | 1 (0.02) | 19 (0.04) | 13 (0.12) * | 10 (0.04) | 6 (0.10) |
| **Multiple myeloma** | 25 (0.17) | 6 (0.16) | 43 (0.17) | 12 (0.19) | 124 (0.29) | 43 (0.40) | 93 (0.40) | 28 (0.48) |
| **Leukemia** | 46 (0.32) | 13 (0.36) | 75 (0.30) | 28 (0.45) | 176 (0.41) | 74 (0.70) * | 142 (0.61) | 35 (0.60) |
| **Mesothelioma** | 9 (0.06) | 3 (0.08) | 20 (0.08) | 5 (0.08) | 57 (0.13) | 13 (0.12) | 61 (0.26) | 17 (0.29) |
| **Breast cancer** | 330 (4.13) | 52 (3.77) | 552 (4.02) | 78 (3.69) | 1032 (4.49) | 158 (4.16) | 556 (4.56) | 108 (4.78) |
| **Cervical cancer** | 4 (0.05) | 1 (0.07) | 10 (0.07) | 1 (0.05) | 16 (0.07) | 7 (0.18) | 13 (0.11) | 5 (0.22) |
| **Endometrial cancer** | 51 (0.64) | 12 (0.87) | 87 (0.63) | 27 (1.28) * | 185 (0.80) | 64 (1.69) * | 119 (0.98) | 35 (1.55) * |
| **Ovarian cancer** | 42 (0.53) | 6 (0.44) | 72 (0.52) | 13 (0.62) | 152 (0.66) | 22 (0.58) | 104 (0.85) | 20 (0.88) |
| **Prostate cancer** | 302 (4.58) | 61 (2.69) * | 533 (4.79) | 138 (3.36) * | 1527 (7.82) | 396 (5.79) * | 1118 (10.06) | 274 (7.69) * |

Data are presented as N (%).

*: represents that there is statistical significance in comparing the difference of cancer incidence between diabetes and non-diabetes at different diagnosis ages by χ2 test.

**Supplementary Table 4. The distribution of cancer mortality in diabetes and non-diabetes at different ages of diagnosis.**

|  | **≤ 40 years** | | **41-50 years** | | **51-60 years** | | **> 60 years** | |
| --- | --- | --- | --- | --- | --- | --- | --- | --- |
|  | **Non-diabetes, n (%)** | **Diabetes, n (%)** | **Non-diabetes, n (%)** | **Diabetes, n (%)** | **Non-diabetes, n (%)** | **Diabetes, n (%)** | **Non-diabetes, n (%)** | **Diabetes, n (%)** |
| **Overall cancer** | 372 (2.55) | 118 (3.24) * | 603 (2.42) | 262 (4.21) * | 1711 (4.02) | 709 (6.67) * | 1351 (5.80) | 497 (8.53) * |
| **Oral cancer** | 9 (0.06) | 1 (0.03) | 10 (0.04) | 4 (0.06) | 31 (0.07) | 8 (0.08) | 24 (0.10) | 4 (0.07) |
| **Esophageal cancer** | 16 (0.11) | 9 (0.25) * | 27 (0.11) | 16 (0.26) * | 75 (0.18) | 57 (0.54) * | 63 (0.27) | 19 (0.33) |
| **Gastric cancer** | 11 (0.08) | 0 (0.00) | 15 (0.06) | 7 (0.11) | 41 (0.10) | 19 (0.18) * | 36 (0.15) | 19 (0.33) * |
| **Colorectal cancer** | 34 (0.23) | 14 (0.38) | 57 (0.23) | 23 (0.37) | 183 (0.43) | 63 (0.59) * | 136 (0.58) | 48 (0.82) * |
| **Liver cancer** | 14 (0.10) | 7 (0.19) | 21 (0.08) | 37 (0.60) * | 54 (0.13) | 59 (0.55) * | 41 (0.18) | 40 (0.69) * |
| **Pancreatic cancer** | 34 (0.23) | 12 (0.33) | 51 (0.21) | 22 (0.35) * | 128 (0.30) | 61 (0.57) * | 88 (0.38) | 61 (1.05) * |
| **Lung cancer** | 64 (0.44) | 21 (0.58) | 109 (0.44) | 43 (0.69) * | 347 (0.82) | 138 (1.30) * | 260 (1.12) | 80 (1.37) |
| **Bone cancer** | / | / | 1 (0.00) | 1 (0.02) | / | / | / | / |
| **Malignant melanoma of skin** | 2 (0.01) | 0 (0.00) | 5 (0.02) | 5 (0.08) * | 24 (0.06) | 7 (0.07) | 16 (0.07) | 8 (0.14) |
| **Renal cancer** | 13 (0.09) | 2 (0.05) | 17 (0.07) | 7 (0.11) | 59 (0.14) | 27 (0.25) * | 30 (0.25) | 19 (0.57) * |
| **Bladder cancer** | 8 (0.05) | 7 (0.19) * | 11 (0.04) | 9 (0.14) * | 43 (0.10) | 18 (0.17) | 2 (0.02) | 17 (0.04) |
| **Brain cancer** | 24 (0.16) | 6 (0.16) | 38 (0.15) | 7 (0.11) | 87 (0.20) | 18 (0.17) | 43 (0.18) | 19 (0.33) * |
| **Thyroid cancer** | / | / | 1 (0.00) | 0 (0.00) | 4 (0.01) | 2 (0.02) | 39 (0.17) | 17 (0.29) |
| **Non-Hodgkin lymphoma** | 14 (0.10) | 6 (0.16) | 21 (0.08) | 10 (0.16) | 73 (0.17) | 19 (0.18) | 52 (0.22) | 15 (0.26) |
| **Hodgkin lymphoma** | 0 (0.00) | 1 (0.03) | 3 (0.01) | 0 (0.00) | 5 (0.01) | 3 (0.03) | 2 (0.01) | 0 (0.00) |
| **Multiple myeloma** | 6 (0.04) | 1 (0.03) | 9 (0.04) | 3 (0.05) | 36 (0.08) | 13 (0.12) | 62 (0.27) | 21 (0.36) |
| **Leukemia** | 14 (0.10) | 3 (0.08) | 19 (0.08) | 6 (0.10) | 49 (0.12) | 26 (0.24) * | 3 (0.01) | 2 (0.03) |
| **Mesothelioma** | 7 (0.05) | 2 (0.05) | 15 (0.06) | 3 (0.05) | 44 (0.10) | 7 (0.07) | 32 (0.14) | 11 (0.19) |
| **Breast cancer** | 15 (0.19) | 8 (0.58) * | 24 (0.17) | 8 (0.38) | 56 (0.24) | 18 (0.47) * | 30 (0.25) | 19 (0.57) * |
| **Cervical cancer** | 1 (0.01) | 0 (0.00) | 2 (0.01) | 0 (0.00) | 2 (0.01) | 0 (0.00) | 2 (0.02) | 17 (0.04) |
| **Endometrial cancer** | 3 (0.04) | 0 (0.00) | 7 (0.05) | 3 (0.14) | 24 (0.10) | 14 (0.37) * | 16 (0.13) | 3 (0.13) |
| **Ovarian cancer** | 21 (0.26) | 2 (0.15) | 26 (0.19) | 8 (0.38) | 67 (0.29) | 14 (0.37) | 46 (0.38) | 10 (0.44) |
| **Prostate cancer** | 21 (0.32) | 3 (0.13) | 36 (0.32) | 16 (0.39) | 119 (0.61) | 52 (0.76) | 115 (1.04) | 39 (1.09) |

Data are presented as N (%).

*: represents that there is statistical significance in comparing the difference of cancer mortality between diabetes and non-diabetes at different diagnosis ages by χ2 test.

**Supplementary Table 5. The relationship between diabetes at different diagnostic ages and cancer incidence risk.**

|  | **Age at diagnosis of diabetes** | | | |
| --- | --- | --- | --- | --- |
|  | **≤ 40 y, HR (95% CI)** | **41-50 y, HR (95% CI)** | **51-60 y, HR (95% CI)** | **> 60 y, HR (95% CI)** |
| **Overall cancer** | 0.996 (0.884-1.122) | 1.078 (0.983-1.181) | 1.088 (1.029-1.152) * | 1.030 (0.963-1.102) |
| **Digestive system cancers** | 1.217 (0.950-1.559) | 1.280 (1.059-1.546) * | 1.442 (1.288-1.614) * | 1.297 (1.136-1.481) * |
| **Oral cancer** | 0.705 (0.277-1.795) | 2.117 (1.250-3.585) * | 0.869 (0.583-1.295) | 0.806 (0.479-1.357) |
| **Esophageal cancer** | 1.157 (0.584-2.294) | 0.935 (0.563-1.555) | 1.605 (1.197-2.152) * | 1.090 (0.738-1.610) |
| **Gastric cancer** | 1.346 (0.621-2.916) | 1.457 (0.813-2.610) | 1.600 (1.133-2.258) * | 1.179 (0.790-1.761) |
| **Colorectal cancer** | 1.224 (0.876-1.712) | 1.081 (0.828-1.413) | 1.190 (1.020-1.388) * | 1.017 (0.848-1.220) |
| **Liver cancer** | 1.251 (0.588-2.662) | 3.142 (1.911-5.169) * | 2.416 (1.743-3.349) * | 2.484 (1.700-3.631) * |
| **Pancreatic cancer** | 1.137 (0.608-2.127) | 1.128 (0.672-1.892) | 1.642 (1.222-2.205) * | 2.154 (1.556-2.981) * |
| **Lung cancer** | 1.101 (0.736-1.648) | 1.045 (0.758-1.440) | 1.271 (1.072-1.508) * | 1.114 (0.910-1.364) |
| **Bone cancer** | 2.467 (0.663-9.174) | 0.659 (0.162-2.687) | 0.792 (0.237-2.646) | 0.682 (0.146-3.175) |
| **Malignant melanoma of skin** | 0.757 (0.429-1.336) | 1.152 (0.786-1.688) | 0.909 (0.706-1.169) | 0.830 (0.601-1.146) |
| **Renal cancer** | 0.772 (0.408-1.460) | 1.528 (1.003-2.328) * | 1.201 (0.915-1.576) | 0.830 (0.577-1.193) |
| **Bladder cancer** | 1.923 (1.224-3.021) * | 1.169 (0.793-1.723) | 1.306 (1.055-1.616) * | 1.288 (1.013-1.636) * |
| **Brain cancer** | 0.739 (0.306-1.785) | 0.755 (0.367-1.551) | 0.761 (0.469-1.235) | 1.140 (0.657-1.977) |
| **Thyroid cancer** | 0.681 (0.209-2.225) | 0.940 (0.407-2.170) | 1.023 (0.476-2.200) | 2.051 (0.890-4.727) |
| **Non-Hodgkin lymphoma** | 1.286 (0.762-2.169) | 1.019 (0.660-1.572) | 0.807 (0.603-1.080) | 0.784 (0.559-1.100) |
| **Hodgkin lymphoma** | / | 0.332 (0.035-3.134) | 2.025 (0.911-4.501) | 1.552 (0.508-4.747) |
| **Multiple myeloma** | 1.015 (0.391-2.640) | 1.054 (0.509-2.183) | 1.232 (0.837-1.813) | 1.195 (0.759-1.881) |
| **Leukemia** | 0.925 (0.479-1.790) | 1.147 (0.699-1.883) | 1.692 (1.249-2.292) * | 0.962 (0.650-1.424) |
| **Mesothelioma** | 0.823 (0.198-3.414) | 0.604 (0.205-1.776) | 0.636 (0.335-1.205) | 0.942 (0.534-1.659) |
| **Breast cancer** | 0.954 (0.698-1.303) | 0.916 (0.703-1.193) | 0.907 (0.755-1.089) | 1.078 (0.864-1.345) |
| **Cervical cancer** | / | 0.496 (0.055-4.483) | 1.828 (0.671-4.974) | 1.285 (0.396-4.169) |
| **Endometrial cancer** | 1.210 (0.613-2.391) | 1.740 (1.055-2.870) * | 1.388 (1.011-1.905) * | 1.094 (0.724-1.652) |
| **Ovarian cancer** | 1.110 (0.455-2.709) | 1.780 (0.909-3.487) | 0.982 (0.603-1.599) | 1.074 (0.644-1.793) |
| **Prostate cancer** | 0.668 (0.499-0.894) * | 0.781 (0.634-0.962) * | 0.811 (0.720-0.914) * | 0.837 (0.727-0.964) * |

The reference for different age of diabetes diagnosis was the matched control.

*: represents *P*<0.05.

/: HR was not calculated due to small sample size.

Multivariable analysis was adjusted for sex, race, income, education, alcohol consumption, physical activity, smoking, body mass index, diet score, family history of cancer, No. of long-term conditions.

Abbreviations: CI, confidence interval; HR, hazard ratio.

**Supplementary Table 6. The relationship between diabetes at different diagnostic ages and cancer mortality risk.**

|  | **Age at diagnosis of diabetes** | | | |
| --- | --- | --- | --- | --- |
|  | **≤ 40 y, HR (95% CI)** | **41-50 y, HR (95% CI)** | **51-60 y, HR (95% CI)** | **> 60 y, HR (95% CI)** |
| **Overall cancer** | 0.924 (0.724-1.180) | 1.149 (0.958-1.377) | 1.276 (1.151-1.416) * | 1.255 (1.119-1.406) * |
| **Digestive system cancers** | 1.070 (0.698-1.641) | 1.345 (0.989-1.829) | 1.680 (1.401-2.015) * | 1.716 (1.407-2.093) * |
| **Oral cancer** | 0.128 (0.009-1.789) | 1.180 (0.280-4.983) | 0.708 (0.285-1.758) | 0.494 (0.159-1.539) |
| **Esophageal cancer** | 2.448 (0.880-6.808) | 1.665 (0.758-3.655) | 2.037 (1.343-3.089) * | 0.934 (0.537-1.625) |
| **Gastric cancer** | / | 0.711 (0.245-2.068) | 1.375 (0.725-2.609) | 1.378 (0.748-2.537) |
| **Colorectal cancer** | 1.061 (0.502-2.239) | 0.968 (0.538-1.743) | 1.215 (0.868-1.701) | 1.286 (0.891-1.856) |
| **Liver cancer** | 1.524 (0.484-4.792) | 3.363 (1.688-6.698) * | 3.269 (2.073-5.154) * | 2.976 (1.807-4.903) * |
| **Pancreatic cancer** | 0.937 (0.426-2.064) | 1.011 (0.542-1.888) | 1.519 (1.057-2.183) * | 2.603 (1.789-3.788) * |
| **Lung cancer** | 1.421 (0.785-2.574) | 1.245 (0.798-1.943) | 1.175 (0.930-1.485) | 0.937 (0.712-1.234) |
| **Bone cancer** | / | / | / | / |
| **Malignant melanoma of skin** | / | / | 0.925 (0.356-2.402) | 1.986 (0.778-5.070) |
| **Renal cancer** | / | 0.610 (0.222-1.677) | 0.886 (0.524-1.496) | 1.147 (0.635-2.073) |
| **Bladder cancer** | 1.944 (0.572-6.610) | 1.507 (0.515-4.410) | 1.833 (0.951-3.535) | 1.817 (0.960-3.438) |
| **Brain cancer** | 0.722 (0.252-2.068) | 0.736 (0.287-1.883) | 0.862 (0.484-1.534) | 1.495 (0.797-2.802) |
| **Thyroid cancer** | / | / | / | / |
| **Non-Hodgkin lymphoma** | 1.274 (0.414-3.923) | 1.268 (0.500-3.217) | 0.855 (0.478-1.530) | 1.218 (0.705-2.103) |
| **Hodgkin lymphoma** | / | / | 0.742 (0.151-3.649) | 0.637 (0.065-6.219) |
| **Multiple myeloma** | / | 1.282 (0.245-6.694) | 0.877 (0.427-1.802) | 1.136 (0.535-2.413) |
| **Leukemia** | 0.707 (0.178-2.806) | 1.044 (0.336-3.245) | 1.834 (1.041-3.229) * | 1.507 (0.851-2.667) |
| **Mesothelioma** | 1.258 (0.168-9.413) | 0.465 (0.105-2.067) | 0.425 (0.178-1.011) | 0.766 (0.381-1.540) |
| **Breast cancer** | 1.470 (0.502-4.305) | 1.217 (0.445-3.325) | 1.240 (0.660-2.330) | 1.653 (0.787-3.470) |
| **Cervical cancer** | / | / | / | / |
| **Endometrial cancer** | / | 2.601 (0.398-17.010) | 2.436 (1.079-5.501) * | 0.660 (0.174-2.502) |
| **Ovarian cancer** | 0.470 (0.099-2.239) | 1.943 (0.713-5.290) | 1.155 (0.588-2.268) | 1.052 (0.492-2.251) |
| **Prostate cancer** | 0.390 (0.103-1.484) | 0.931 (0.456-1.899) | 1.209 (0.833-1.755) | 1.314 (0.882-1.958) |

The reference for different age of diabetes diagnosis was the matched control without diabetes.

*: represents *P*<0.05.

/: HR was not calculated due to small sample size.

Multivariable analysis was adjusted for sex, race, income, education, alcohol consumption, physical activity, smoking, body mass index, diet score, family history of cancer, No. of long-term conditions, antihypertensive and lipid-lowering drugs.

Abbreviations: CI, confidence interval; HR, hazard ratio.

**Supplementary Table 7. Association between diabetes at different diagnostic ages and cancer incidence risk after excluding participants with outcome events within the first year of follow-up.**

|  | **Age at diagnosis of diabetes** | | | |
| --- | --- | --- | --- | --- |
|  | **≤ 40 y, HR (95% CI)** | **41-50 y, HR (95% CI)** | **51-60 y, HR (95% CI)** | **> 60 y, HR (95% CI)** |
| **Overall cancer** | 0.991 (0.876-1.121) | 1.076 (0.979-1.184) | 1.091 (1.029-1.156) * | 1.027 (0.958-1.102) |
| **Digestive system cancers** | 1.225 (0.948-1.582) | 1.308 (1.078-1.588) * | 1.449 (1.291-1.627) * | 1.298 (1.133-1.488) * |
| **Oral cancer** | 0.467 (0.153-1.422) | 2.040 (1.192-3.493) * | 0.887 (0.593-1.326) | 0.768 (0.444-1.329) |
| **Esophageal cancer** | 1.296 (0.645-2.605) | 0.985 (0.579-1.675) | 1.635 (1.209-2.213) * | 0.992 (0.660-1.492) |
| **Gastric cancer** | 1.333 (0.585-3.033) | 1.506 (0.822-2.761) | 1.555 (1.089-2.219) * | 1.172 (0.780-1.760) |
| **Colorectal cancer** | 1.229 (0.869-1.739) | 1.086 (0.824-1.432) | 1.209 (1.032-1.417) * | 1.026 (0.848-1.241) |
| **Liver cancer** | 1.150 (0.509-2.597) | 3.493 (2.064-5.912) * | 2.621 (1.875-3.663) * | 2.723 (1.834-4.044) * |
| **Pancreatic cancer** | 1.208 (0.646-2.259) | 1.107 (0.651-1.883) | 1.555 (1.146-2.108) * | 1.959 (1.395-2.752) * |
| **Lung cancer** | 0.915 (0.594-1.410) | 0.958 (0.683-1.343) | 1.262 (1.060-1.503) * | 1.074 (0.871-1.325) |
| **Bone cancer** | 3.025 (0.785-11.665) | 0.632 (0.147-2.711) | 0.947 (0.272-3.304) | 0.377 (0.045-3.153) |
| **Malignant melanoma of skin** | 0.755 (0.420-1.357) | 1.159 (0.781-1.718) | 0.909 (0.702-1.176) | 0.816 (0.583-1.143) |
| **Renal cancer** | 0.928 (0.486-1.774) | 1.558 (1.007-2.411) * | 1.213 (0.917-1.605) | 0.869 (0.601-1.257) |
| **Bladder cancer** | 1.677 (1.040-2.705) * | 1.123 (0.746-1.689) | 1.259 (1.002-1.581) * | 1.294 (1.007-1.664) * |
| **Brain cancer** | 0.686 (0.267-1.763) | 0.647 (0.296-1.415) | 0.749 (0.453-1.240) | 1.058 (0.581-1.928) |
| **Thyroid cancer** | 0.788 (0.243-2.562) | 1.014 (0.420-2.448) | 1.048 (0.484-2.270) | 1.874 (0.787-4.461) |
| **Non-Hodgkin lymphoma** | 1.212 (0.704-2.086) | 1.049 (0.677-1.623) | 0.779 (0.576-1.053) | 0.774 (0.546-1.099) |
| **Hodgkin lymphoma** | / | 0.331 (0.035-3.127) | 1.840 (0.758-4.464) | 2.234 (0.592-8.436) |
| **Multiple myeloma** | 1.017 (0.391-2.645) | 0.922 (0.424-2.003) | 1.229 (0.828-1.825) | 1.245 (0.783-1.980) |
| **Leukemia** | 0.937 (0.472-1.861) | 1.217 (0.732-2.023) | 1.743 (1.271-2.389) * | 0.999 (0.669-1.490) |
| **Mesothelioma** | 0.601 (0.113-3.200) | 0.627 (0.210-1.874) | 0.621 (0.311-1.240) | 0.940 (0.514-1.716) |
| **Breast cancer** | 0.994 (0.720-1.372) | 0.888 (0.669-1.180) | 0.908 (0.749-1.101) | 1.045 (0.824-1.325) |
| **Cervical cancer** | / | 0.578 (0.062-5.415) | 1.633 (0.571-4.674) | 1.153 (0.331-4.018) |
| **Endometrial cancer** | 1.307 (0.658-2.598) | 1.612 (0.953-2.728) | 1.398 (1.007-1.939) * | 1.052 (0.681-1.623) |
| **Ovarian cancer** | 1.208 (0.456-3.204) | 1.961 (0.969-3.966) | 1.097 (0.669-1.800) | 0.948 (0.539-1.669) |
| **Prostate cancer** | 0.692 (0.512-0.934) * | 0.777 (0.627-0.964) * | 0.821 (0.725-0.928) * | 0.849 (0.734-0.983) * |

The reference for different age of diabetes diagnosis was the matched control.

*: represents *P*<0.05.

/: HR was not calculated due to small sample size.

Multivariable analysis was adjusted for sex, race, income, education, alcohol consumption, physical activity, smoking, body mass index, diet score, family history of cancer, No. of long-term conditions.

Abbreviations: CI, confidence interval; HR, hazard ratio.

**Supplementary Table 8. Association between diabetes at different diagnostic ages and cancer mortality risk after excluding participants**

**with outcome events within the first year of follow-up.**

|  | **Age at diagnosis of diabetes** | | | |
| --- | --- | --- | --- | --- |
|  | **≤ 40 y, HR (95% CI)** | **41-50 y, HR (95% CI)** | **51-60 y, HR (95% CI)** | **> 60 y, HR (95% CI)** |
| **Overall cancer** | 0.926 (0.723-1.186) | 1.148 (0.956-1.379) | 1.270 (1.144-1.410) * | 1.250 (1.114-1.402) * |
| **Digestive system cancers** | 1.099 (0.708-1.706) | 1.389 (1.017-1.898) * | 1.697 (1.413-2.038) * | 1.715 (1.403-2.096) * |
| **Oral cancer** | 0.128 (0.009-1.789) | 1.180 (0.280-4.983) | 0.708 (0.285-1.758) | 0.494 (0.159-1.539) |
| **Esophageal cancer** | 3.171 (1.102-9.130) * | 1.906 (0.857-4.238) | 2.034 (1.337-3.095) * | 0.934 (0.537-1.625) |
| **Gastric cancer** | / | 0.685 (0.233-2.012) | 1.375 (0.725-2.609) | 1.378 (0.748-2.537) |
| **Colorectal cancer** | 1.000 (0.466-2.146) | 0.968 (0.538-1.743) | 1.228 (0.876-1.720) | 1.316 (0.910-1.901) |
| **Liver cancer** | 1.548 (0.444-5.402) | 3.487 (1.705-7.131) * | 3.442 (2.171-5.457) * | 3.158 (1.903-5.239) * |
| **Pancreatic cancer** | 1.028 (0.463-2.281) | 1.065 (0.563-2.017) | 1.519 (1.049-2.200) * | 2.501 (1.704-3.670) * |
| **Lung cancer** | 1.388 (0.758-2.541) | 1.187 (0.752-1.874) | 1.150 (0.908-1.457) | 0.922 (0.699-1.217) |
| **Bone cancer** | / | / | / | / |
| **Malignant melanoma of skin** | / | / | 0.925 (0.356-2.402) | 1.986 (0.778-5.070) |
| **Renal cancer** | / | 0.610 (0.222-1.677) | 0.856 (0.503-1.456) | 1.147 (0.635-2.073) |
| **Bladder cancer** | 1.944 (0.572-6.610) | 1.507 (0.515-4.410) | 1.833 (0.951-3.535) | 1.817 (0.960-3.438) |
| **Brain cancer** | 0.730 (0.254-2.099) | 0.746 (0.290-1.915) | 0.866 (0.479-1.564) | 1.431 (0.749-2.734) |
| **Thyroid cancer** | / | / | / | / |
| **Non-Hodgkin lymphoma** | 1.274 (0.414-3.923) | 1.268 (0.500-3.217) | 0.819 (0.453-1.482) | 1.197 (0.685-2.093) |
| **Hodgkin lymphoma** | / | / | 0.742 (0.151-3.649) | / |
| **Multiple myeloma** | / | 1.282 (0.245-6.694) | 0.877 (0.427-1.802) | 1.136 (0.535-2.413) |
| **Leukemia** | 0.707 (0.178-2.806) | 1.044 (0.336-3.245) | 1.765 (0.996-3.126) | 1.507 (0.851-2.667) |
| **Mesothelioma** | 1.258 (0.168-9.413) | 0.465 (0.105-2.067) | 0.352 (0.129-1.961) | 0.724 (0.351-1.495) |
| **Breast cancer** | 1.470 (0.502-4.305) | 1.217 (0.445-3.325) | 1.240 (0.660-2.330) | 1.653 (0.787-3.470) |
| **Cervical cancer** | / | / | / | / |
| **Endometrial cancer** | / | / | 2.436 (1.079-5.501) * | 0.775 (0.202-2.977) |
| **Ovarian cancer** | 0.470 (0.099-2.239) | 1.943 (0.713-5.290) | 1.155 (0.588-2.268) | 1.052 (0.492-2.251) |
| **Prostate cancer** | 0.390 (0.103-1.484) | 0.931 (0.456-1.899) | 1.217 (0.838-1.767) | 1.322 (0.887-1.971) |

The reference for different age of diabetes diagnosis was the matched control.

*: represents *P*<0.05.

/: HR was not calculated due to small sample size.

Multivariable analysis was adjusted for sex, race, income, education, alcohol consumption, physical activity, smoking, body mass index, diet score, family history of cancer, No. of long-term conditions, antihypertensive and lipid-lowering drugs.

Abbreviations: CI, confidence interval; HR, hazard ratio.

**Supplementary Table 9. Association between diabetes at different diagnostic ages and cancer incidence risk: results from multivariate competing risk model.**

|  | **Age at diagnosis of diabetes** | | | |
| --- | --- | --- | --- | --- |
|  | **≤ 40 y, HR (95% CI)** | **41-50 y, HR (95% CI)** | **51-60 y, HR (95% CI)** | **>60 y, HR (95% CI)** |
| **Overall cancer** | 0.948 (0.840-1.070) | 1.050 (0.957-1.151) | 1.066 (1.008-1.129) * | 1.006 (0.940-1.076) |
| **Digestive system cancers** | 1.155 (0.891-1.498) | 1.238 (1.028-1.490) * | 1.409 (1.256-1.580) * | 1.261 (1.103-1.441) * |
| **Oral cancer** | 0.673 (0.263-1.726) | 2.043 (1.228-3.401) * | 0.839 (0.553-1.273) | 0.780 (0.440-1.384) |
| **Esophageal cancer** | 1.099 (0.523-2.308) | 0.900 (0.564-1.437) | 1.554 (1.164-2.075) * | 1.050 (0.698-1.581) |
| **Gastric cancer** | 1.276 (0.578-2.815) | 1.412 (0.805-2.474) | 1.551 (1.104-2.178) * | 1.141 (0.773-1.683) |
| **Colorectal cancer** | 1.150 (0.813-1.626) | 1.047 (0.802-1.366) | 1.157 (0.989-1.354) | 0.987 (0.822-1.187) |
| **Liver cancer** | 1.150 (0.563-2.348) | 3.003 (1.818-4.959) * | 2.338 (1.653-3.307) * | 2.396 (1.610-3.565) * |
| **Pancreatic cancer** | 1.080 (0.562-2.076) | 1.085 (0.656-1.793) | 1.597 (1.190-2.143) * | 2.101 (1.522-2.902) * |
| **Lung cancer** | 1.004 (0.662-1.523) | 1.004 (0.728-1.386) | 1.226 (1.034-1.455) * | 1.073 (0.876-1.314) |
| **Bone cancer** | 2.421 (0.608-9.639) | 0.639 (0.218-1.874) | 0.774 (0.292-2.047) | 0.671 (0.126-3.584) |
| **Malignant melanoma of skin** | 0.722 (0.410-1.272) | 1.123 (0.768-1.640) | 0.884 (0.691-1.132) | 0.808 (0.588-1.111) |
| **Renal cancer** | 0.723 (0.388-1.349) | 1.479 (0.952-2.298) | 1.161 (0.873-1.545) | 0.802 (0.564-1.141) |
| **Bladder cancer** | 1.828 (1.132-2.953) * | 1.128 (0.755-1.685) | 1.269 (1.021-1.578) * | 1.251 (0.981-1.594) |
| **Brain cancer** | 0.702 (0.282-1.746) | 0.724 (0.341-1.541) | 0.736 (0.464-1.170) | 1.113 (0.637-1.947) |
| **Thyroid cancer** | 0.641 (0.167-2.459) | 0.912 (0.399-2.086) | 1.000 (0.477-2.100) | 2.015 (0.977-4.159) |
| **Non-Hodgkin lymphoma** | 1.210 (0.667-2.198) | 0.984 (0.632-1.532) | 0.784 (0.589-1.044) | 0.763 (0.543-1.072) |
| **Hodgkin lymphoma** | / | / | 1.979 (0.849-4.613) | 1.522 (0.488-4.749) |
| **Multiple myeloma** | 0.951 (0.398-2.272) | 1.023 (0.522-2.003) | 1.193 (0.824-1.728) | 1.165 (0.732-1.855) |
| **Leukemia** | 0.872 (0.435-1.746) | 1.107 (0.690-1.776) | 1.647 (1.214-2.234) * | 0.934 (0.633-1.377) |
| **Mesothelioma** | 0.764 (0.180-3.238) | 0.581 (0.178-1.894) | 0.614 (0.319-1.180) | 0.917 (0.527-1.598) |
| **Breast cancer** | 0.926 (0.672-1.276) | 0.903 (0.691-1.179) | 0.895 (0.747-1.072) | 1.062 (0.845-1.336) |
| **Cervical cancer** | / | / | 1.809 (0.607-5.388) | 1.254 (0.454-3.465) |
| **Endometrial cancer** | 1.172 (0.623-2.203) | 1.715 (1.045-2.815) * | 1.369 (1.004-1.867) * | 1.074 (0.715-1.613) |
| **Ovarian cancer** | 1.076 (0.510-2.272) | 1.743 (0.910-3.338) | 0.966 (0.587-1.589) | 1.059 (0.658-1.705) |
| **Prostate cancer** | 0.635 (0.469-0.860) | 0.754 (0.609-0.934) | 0.789 (0.700-0.890) | 0.816 (0.707-0.941) |

The reference for different age of diabetes diagnosis was the matched control.

*: represents *P*<0.05.

/: HR was not calculated due to small sample size.

Multivariable analysis was adjusted for sex, race, income, education, alcohol consumption, physical activity, smoking, body mass index, diet score, family history of cancer, No. of long-term conditions, antihypertensive and lipid-lowering drugs.

Abbreviations: CI, confidence interval; HR, hazard ratio.

**Supplementary Table 10. Association between diabetes at different diagnostic ages and cancer mortality risk: results from multivariate competing risk model.**

|  | **Age at diagnosis of diabetes** | | | |
| --- | --- | --- | --- | --- |
|  | **≤ 40 y, HR (95% CI)** | **41-50 y, HR (95% CI)** | **51-60 y, HR (95% CI)** | **>60 y, HR (95% CI)** |
| **Overall cancer** | 0.864 (0.671-1.111) | 1.111 (0.931-1.326) | 1.246 (1.123-1.383) * | 1.222 (1.091-1.370) * |
| **Digestive system cancers** | 1.024 0.655 1.601 | 1.299 0.972 1.736 | 1.653 1.375 1.987 | 1.670 1.364 2.044 |
| **Oral cancer** | 0.135 (0.011-1.671) | 1.102 (0.245-4.959) | 0.666 (0.251-1.766) | 0.463 (0.141-1.524) |
| **Esophageal cancer** | 2.332 (0.695-7.821) | 1.599 (0.762-3.358) | 1.962 (1.308-2.942) * | 0.897 (0.507-1.586) |
| **Gastric cancer** | / | 0.691 (0.230-2.076) | 1.333 (0.710-2.501) | 1.313 (0.755-2.284) |
| **Colorectal cancer** | 0.980 (0.503-1.908) | 0.938 (0.520-1.693) | 1.178 (0.835-1.662) | 1.246 (0.859-1.807) |
| **Liver cancer** | 1.436 (0.487-4.236) | 3.204 (1.751-5.865) * | 3.176 (1.960-5.147) * | 2.859 (1.677-4.873) * |
| **Pancreatic cancer** | 0.879 (0.389-1.989) | 0.974 (0.553-1.718) | 1.480 (1.034-2.119) * | 2.549 (1.754-3.704) * |
| **Lung cancer** | 1.328 (0.727-2.427) | 1.210 (0.790-1.855) | 1.143 (0.905-1.445) | 0.906 (0.688-1.192) |
| **Bone cancer** | / | / | / | / |
| **Malignant melanoma of skin** | / | / | 0.897 (0.352-2.286) | 1.897 (0.721-4.992) |
| **Renal cancer** | / | 0.585 (0.189-1.807) | 0.855 (0.506-1.446) | 1.101 (0.606-2.003) |
| **Bladder cancer** | 1.710 (0.575-5.087) | 1.425 (0.611-3.327) | 1.779 (0.881-3.594) | 1.744 (0.893-3.408) |
| **Brain cancer** | 0.674 (0.224-2.024) | 0.717 (0.267-1.924) | 0.834 (0.483-1.440) | 1.461 (0.787-2.711) |
| **Thyroid cancer** | / | / | / | / |
| **non-Hodgkin lymphoma** | 1.164 (0.334-4.051) | 1.211 (0.498-2.943) | 0.821 (0.469-1.438) | 1.177 (0.691-2.006) |
| **Hodgkin lymphoma** | / | / | 0.715 (0.133-3.846) | 0.607 (0.206-1.789) |
| **Multiple myeloma** | / | 1.225 (0.297-5.047) | 0.845 (0.412-1.732) | 1.104 (0.546-2.231) |
| **Leukemia** | 0.661 (0.161-2.715) | 1.013 (0.372-2.760) | 1.785 (1.046-3.046) * | 1.453 (0.855-2.469) |
| **Mesothelioma** | 1.022 (0.155-6.731) | 0.445 (0.094-2.095) | 0.411 (0.175-1.969) | 0.748 (0.373-1.500) |
| **Breast cancer** | 1.451 (0.404-5.210) | 1.198 (0.410-3.501) | 1.215 (0.634-2.326) | 1.604 (0.748-3.436) |
| **Cervical cancer** | / | / | / | / |
| **Endometrial cancer** | / | / | 2.388 (1.093-5.216) * | 0.644 (0.157-2.633) |
| **Ovarian cancer** | 0.445 (0.096-2.060) | 1.892 (0.628-5.694) | 1.132 (0.553-2.317) | 1.044 (0.511-2.135) |
| **Prostate cancer** | 0.357 (0.099-1.283) | 0.886 (0.501-1.568) | 1.157 (0.814-1.644) | 1.259 (0.846-1.875) |

The reference for different age of diabetes diagnosis was the matched control.

*: represents *P*<0.05.

/: HR was not calculated due to small sample size.

Multivariable analysis was adjusted for sex, race, income, education, alcohol consumption, physical activity, smoking, body mass index, diet score, family history of cancer, No. of long-term conditions, antihypertensive and lipid-lowering drugs.

Abbreviations: CI, confidence interval; HR, hazard ratio.


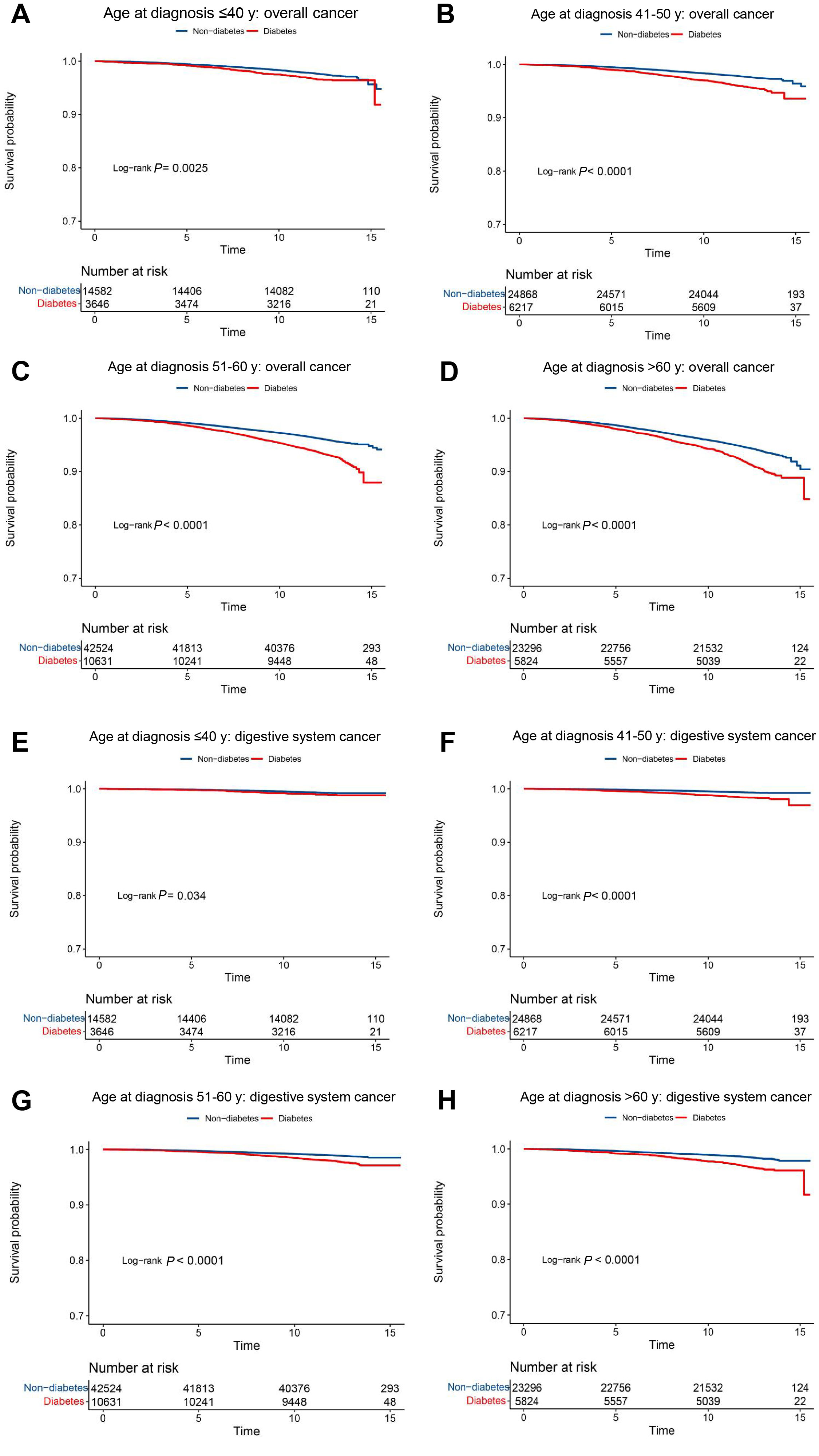


**Supplementary Figure 1. Survival curves of overall cancer and digestive system cancer in different age groups diagnosed with diabetes.**
